# Environmental and Health Impacts of Air Pollution: A Review

**DOI:** 10.3389/fpubh.2020.00014

**Published:** 2020-02-20

**Authors:** Ioannis Manisalidis, Elisavet Stavropoulou, Agathangelos Stavropoulos, Eugenia Bezirtzoglou

**Affiliations:** ^1^Delphis S.A., Kifisia, Greece; ^2^Laboratory of Hygiene and Environmental Protection, Faculty of Medicine, Democritus University of Thrace, Alexandroupolis, Greece; ^3^Centre Hospitalier Universitaire Vaudois (CHUV), Service de Médicine Interne, Lausanne, Switzerland; ^4^School of Social and Political Sciences, University of Glasgow, Glasgow, United Kingdom

**Keywords:** air pollution, environment, health, public health, gas emission, policy

## Abstract

One of our era's greatest scourges is air pollution, on account not only of its impact on climate change but also its impact on public and individual health due to increasing morbidity and mortality. There are many pollutants that are major factors in disease in humans. Among them, Particulate Matter (PM), particles of variable but very small diameter, penetrate the respiratory system via inhalation, causing respiratory and cardiovascular diseases, reproductive and central nervous system dysfunctions, and cancer. Despite the fact that ozone in the stratosphere plays a protective role against ultraviolet irradiation, it is harmful when in high concentration at ground level, also affecting the respiratory and cardiovascular system. Furthermore, nitrogen oxide, sulfur dioxide, Volatile Organic Compounds (VOCs), dioxins, and polycyclic aromatic hydrocarbons (PAHs) are all considered air pollutants that are harmful to humans. Carbon monoxide can even provoke direct poisoning when breathed in at high levels. Heavy metals such as lead, when absorbed into the human body, can lead to direct poisoning or chronic intoxication, depending on exposure. Diseases occurring from the aforementioned substances include principally respiratory problems such as Chronic Obstructive Pulmonary Disease (COPD), asthma, bronchiolitis, and also lung cancer, cardiovascular events, central nervous system dysfunctions, and cutaneous diseases. Last but not least, climate change resulting from environmental pollution affects the geographical distribution of many infectious diseases, as do natural disasters. The only way to tackle this problem is through public awareness coupled with a multidisciplinary approach by scientific experts; national and international organizations must address the emergence of this threat and propose sustainable solutions.

## Approach to the Problem

The interactions between humans and their physical surroundings have been extensively studied, as multiple human activities influence the environment. The environment is a coupling of the biotic (living organisms and microorganisms) and the abiotic (hydrosphere, lithosphere, and atmosphere).

Pollution is defined as the introduction into the environment of substances harmful to humans and other living organisms. Pollutants are harmful solids, liquids, or gases produced in higher than usual concentrations that reduce the quality of our environment.

Human activities have an adverse effect on the environment by polluting the water we drink, the air we breathe, and the soil in which plants grow. Although the industrial revolution was a great success in terms of technology, society, and the provision of multiple services, it also introduced the production of huge quantities of pollutants emitted into the air that are harmful to human health. Without any doubt, the global environmental pollution is considered an international public health issue with multiple facets. Social, economic, and legislative concerns and lifestyle habits are related to this major problem. Clearly, urbanization and industrialization are reaching unprecedented and upsetting proportions worldwide in our era. Anthropogenic air pollution is one of the biggest public health hazards worldwide, given that it accounts for about 9 million deaths per year (1).

Without a doubt, all of the aforementioned are closely associated with climate change, and in the event of danger, the consequences can be severe for mankind (2). Climate changes and the effects of global planetary warming seriously affect multiple ecosystems, causing problems such as food safety issues, ice and iceberg melting, animal extinction, and damage to plants (3, 4).

Air pollution has various health effects. The health of susceptible and sensitive individuals can be impacted even on low air pollution days. Short-term exposure to air pollutants is closely related to COPD (Chronic Obstructive Pulmonary Disease), cough, shortness of breath, wheezing, asthma, respiratory disease, and high rates of hospitalization (a measurement of morbidity).

The long-term effects associated with air pollution are chronic asthma, pulmonary insufficiency, cardiovascular diseases, and cardiovascular mortality. According to a Swedish cohort study, diabetes seems to be induced after long-term air pollution exposure (5). Moreover, air pollution seems to have various malign health effects in early human life, such as respiratory, cardiovascular, mental, and perinatal disorders (3), leading to infant mortality or chronic disease in adult age (6).

National reports have mentioned the increased risk of morbidity and mortality (1). These studies were conducted in many places around the world and show a correlation between daily ranges of particulate matter (PM) concentration and daily mortality. Climate shifts and global planetary warming (3) could aggravate the situation. Besides, increased hospitalization (an index of morbidity) has been registered among the elderly and susceptible individuals for specific reasons. Fine and ultrafine particulate matter seems to be associated with more serious illnesses (6), as it can invade the deepest parts of the airways and more easily reach the bloodstream.

Air pollution mainly affects those living in large urban areas, where road emissions contribute the most to the degradation of air quality. There is also a danger of industrial accidents, where the spread of a toxic fog can be fatal to the populations of the surrounding areas. The dispersion of pollutants is determined by many parameters, most notably atmospheric stability and wind (6).

In developing countries (7), the problem is more serious due to overpopulation and uncontrolled urbanization along with the development of industrialization. This leads to poor air quality, especially in countries with social disparities and a lack of information on sustainable management of the environment. The use of fuels such as wood fuel or solid fuel for domestic needs due to low incomes exposes people to bad-quality, polluted air at home. It is of note that three billion people around the world are using the above sources of energy for their daily heating and cooking needs (8). In developing countries, the women of the household seem to carry the highest risk for disease development due to their longer duration exposure to the indoor air pollution (8, 9). Due to its fast industrial development and overpopulation, China is one of the Asian countries confronting serious air pollution problems (10, 11). The lung cancer mortality observed in China is associated with fine particles (12). As stated already, long-term exposure is associated with deleterious effects on the cardiovascular system (3, 5). However, it is interesting to note that cardiovascular diseases have mostly been observed in developed and high-income countries rather than in the developing low-income countries exposed highly to air pollution (13). Extreme air pollution is recorded in India, where the air quality reaches hazardous levels. New Delhi is one of the more polluted cities in India. Flights in and out of New Delhi International Airport are often canceled due to the reduced visibility associated with air pollution. Pollution is occurring both in urban and rural areas in India due to the fast industrialization, urbanization, and rise in use of motorcycle transportation. Nevertheless, biomass combustion associated with heating and cooking needs and practices is a major source of household air pollution in India and in Nepal (14, 15). There is spatial heterogeneity in India, as areas with diverse climatological conditions and population and education levels generate different indoor air qualities, with higher PM_2.5_ observed in North Indian states (557–601 μg/m^3^) compared to the Southern States (183–214 μg/m^3^) (16, 17). The cold climate of the North Indian areas may be the main reason for this, as longer periods at home and more heating are necessary compared to in the tropical climate of Southern India. Household air pollution in India is associated with major health effects, especially in women and young children, who stay indoors for longer periods. Chronic obstructive respiratory disease (CORD) and lung cancer are mostly observed in women, while acute lower respiratory disease is seen in young children under 5 years of age (18).

Accumulation of air pollution, especially sulfur dioxide and smoke, reaching 1,500 mg/m3, resulted in an increase in the number of deaths (4,000 deaths) in December 1952 in London and in 1963 in New York City (400 deaths) (19). An association of pollution with mortality was reported on the basis of monitoring of outdoor pollution in six US metropolitan cities (20). In every case, it seems that mortality was closely related to the levels of fine, inhalable, and sulfate particles more than with the levels of total particulate pollution, aerosol acidity, sulfur dioxide, or nitrogen dioxide (20).

Furthermore, extremely high levels of pollution are reported in Mexico City and Rio de Janeiro, followed by Milan, Ankara, Melbourne, Tokyo, and Moscow (19).

Based on the magnitude of the public health impact, it is certain that different kinds of interventions should be taken into account. Success and effectiveness in controlling air pollution, specifically at the local level, have been reported. Adequate technological means are applied considering the source and the nature of the emission as well as its impact on health and the environment. The importance of point sources and non-point sources of air pollution control is reported by Schwela and Köth-Jahr (21). Without a doubt, a detailed emission inventory must record all sources in a given area. Beyond considering the above sources and their nature, topography and meteorology should also be considered, as stated previously. Assessment of the control policies and methods is often extrapolated from the local to the regional and then to the global scale. Air pollution may be dispersed and transported from one region to another area located far away. Air pollution management means the reduction to acceptable levels or possible elimination of air pollutants whose presence in the air affects our health or the environmental ecosystem. Private and governmental entities and authorities implement actions to ensure the air quality (22). Air quality standards and guidelines were adopted for the different pollutants by the WHO and EPA as a tool for the management of air quality (1, 23). These standards have to be compared to the emissions inventory standards by causal analysis and dispersion modeling in order to reveal the problematic areas (24). Inventories are generally based on a combination of direct measurements and emissions modeling (24).

As an example, we state here the control measures at the source through the use of catalytic converters in cars. These are devices that turn the pollutants and toxic gases produced from combustion engines into less-toxic pollutants by catalysis through redox reactions (25). In Greece, the use of private cars was restricted by tracking their license plates in order to reduce traffic congestion during rush hour (25).

Concerning industrial emissions, collectors and closed systems can keep the air pollution to the minimal standards imposed by legislation (26).

Current strategies to improve air quality require an estimation of the economic value of the benefits gained from proposed programs. These proposed programs by public authorities, and directives are issued with guidelines to be respected.

In Europe, air quality limit values AQLVs (Air Quality Limit Values) are issued for setting off planning claims (27). In the USA, the NAAQS (National Ambient Air Quality Standards) establish the national air quality limit values (27). While both standards and directives are based on different mechanisms, significant success has been achieved in the reduction of overall emissions and associated health and environmental effects (27). The European Directive identifies geographical areas of risk exposure as monitoring/assessment zones to record the emission sources and levels of air pollution (27), whereas the USA establishes global geographical air quality criteria according to the severity of their air quality problem and records all sources of the pollutants and their precursors (27).

In this vein, funds have been financing, directly or indirectly, projects related to air quality along with the technical infrastructure to maintain good air quality. These plans focus on an inventory of databases from air quality environmental planning awareness campaigns. Moreover, pollution measures of air emissions may be taken for vehicles, machines, and industries in urban areas.

Technological innovation can only be successful if it is able to meet the needs of society. In this sense, technology must reflect the decision-making practices and procedures of those involved in risk assessment and evaluation and act as a facilitator in providing information and assessments to enable decision makers to make the best decisions possible. Summarizing the aforementioned in order to design an effective air quality control strategy, several aspects must be considered: environmental factors and ambient air quality conditions, engineering factors and air pollutant characteristics, and finally, economic operating costs for technological improvement and administrative and legal costs. Considering the economic factor, competitiveness through neoliberal concepts is offering a solution to environmental problems (22).

The development of environmental governance, along with technological progress, has initiated the deployment of a dialogue. Environmental politics has created objections and points of opposition between different political parties, scientists, media, and governmental and non-governmental organizations (22). Radical environmental activism actions and movements have been created (22). The rise of the new information and communication technologies (ICTs) are many times examined as to whether and in which way they have influenced means of communication and social movements such as activism (28). Since the 1990s, the term “digital activism” has been used increasingly and in many different disciplines (29). Nowadays, multiple digital technologies can be used to produce a digital activism outcome on environmental issues. More specifically, devices with online capabilities such as computers or mobile phones are being used as a way to pursue change in political and social affairs (30).

In the present paper, we focus on the sources of environmental pollution in relation to public health and propose some solutions and interventions that may be of interest to environmental legislators and decision makers.

## Sources of Exposure

It is known that the majority of environmental pollutants are emitted through large-scale human activities such as the use of industrial machinery, power-producing stations, combustion engines, and cars. Because these activities are performed at such a large scale, they are by far the major contributors to air pollution, with cars estimated to be responsible for approximately 80% of today's pollution (31). Some other human activities are also influencing our environment to a lesser extent, such as field cultivation techniques, gas stations, fuel tanks heaters, and cleaning procedures (32), as well as several natural sources, such as volcanic and soil eruptions and forest fires.

The classification of air pollutants is based mainly on the sources producing pollution. Therefore, it is worth mentioning the four main sources, following the classification system: Major sources, Area sources, Mobile sources, and Natural sources.

*Major sources* include the emission of pollutants from power stations, refineries, and petrochemicals, the chemical and fertilizer industries, metallurgical and other industrial plants, and, finally, municipal incineration.

*Indoor area sources* include domestic cleaning activities, dry cleaners, printing shops, and petrol stations.

*Mobile sources* include automobiles, cars, railways, airways, and other types of vehicles.

Finally, *natural sources* include, as stated previously, physical disasters (33) such as forest fire, volcanic erosion, dust storms, and agricultural burning.

However, many classification systems have been proposed. Another type of classification is a grouping according to the recipient of the pollution, as follows:

*Air pollution* is determined as the presence of pollutants in the air in large quantities for long periods. Air pollutants are dispersed particles, hydrocarbons, CO, CO_2_, NO, NO_2_, SO_3_, etc.

*Water pollution* is organic and inorganic charge and biological charge (10) at high levels that affect the water quality (34, 35).

*Soil pollution* occurs through the release of chemicals or the disposal of wastes, such as heavy metals, hydrocarbons, and pesticides.

Air pollution can influence the quality of soil and water bodies by polluting precipitation, falling into water and soil environments (34, 36). Notably, the chemistry of the soil can be amended due to acid precipitation by affecting plants, cultures, and water quality (37). Moreover, movement of heavy metals is favored by soil acidity, and metals are so then moving into the watery environment. It is known that heavy metals such as aluminum are noxious to wildlife and fishes. Soil quality seems to be of importance, as soils with low calcium carbonate levels are at increased jeopardy from acid rain. Over and above rain, snow and particulate matter drip into watery ' bodies (36, 38).

Lastly, pollution is classified following type of origin:

*Radioactive and nuclear pollution*, releasing radioactive and nuclear pollutants into water, air, and soil during nuclear explosions and accidents, from nuclear weapons, and through handling or disposal of radioactive sewage.

Radioactive materials can contaminate surface water bodies and, being noxious to the environment, plants, animals, and humans. It is known that several radioactive substances such as radium and uranium concentrate in the bones and can cause cancers (38, 39).

*Noise pollution* is produced by machines, vehicles, traffic noises, and musical installations that are harmful to our hearing.

The World Health Organization introduced the term DALYs. The DALYs for a disease or health condition is defined as the sum of the Years of Life Lost (YLL) due to premature mortality in the population and the Years Lost due to Disability (YLD) for people living with the health condition or its consequences (39). In Europe, air pollution is the main cause of disability-adjusted life years lost (DALYs), followed by noise pollution. The potential relationships of noise and air pollution with health have been studied (40). The study found that DALYs related to noise were more important than those related to air pollution, as the effects of environmental noise on cardiovascular disease were independent of air pollution (40). Environmental noise should be counted as an independent public health risk (40).

*Environmental pollution* occurs when changes in the physical, chemical, or biological constituents of the environment (air masses, temperature, climate, etc.) are produced.

Pollutants harm our environment either by increasing levels above normal or by introducing harmful toxic substances. Primary pollutants are directly produced from the above sources, and secondary pollutants are emitted as by-products of the primary ones. Pollutants can be biodegradable or non-biodegradable and of natural origin or anthropogenic, as stated previously. Moreover, their origin can be a unique source (point-source) or dispersed sources.

Pollutants have differences in physical and chemical properties, explaining the discrepancy in their capacity for producing toxic effects. As an example, we state here that aerosol compounds (41–43) have a greater toxicity than gaseous compounds due to their tiny size (solid or liquid) in the atmosphere; they have a greater penetration capacity. Gaseous compounds are eliminated more easily by our respiratory system (41). These particles are able to damage lungs and can even enter the bloodstream (41), leading to the premature deaths of millions of people yearly. Moreover, the aerosol acidity ([H+]) seems to considerably enhance the production of secondary organic aerosols (SOA), but this last aspect is not supported by other scientific teams (38).

## Climate and Pollution

Air pollution and climate change are closely related. Climate is the other side of the same coin that reduces the quality of our Earth (44). Pollutants such as black carbon, methane, tropospheric ozone, and aerosols affect the amount of incoming sunlight. As a result, the temperature of the Earth is increasing, resulting in the melting of ice, icebergs, and glaciers.

In this vein, climatic changes will affect the incidence and prevalence of both residual and imported infections in Europe. Climate and weather affect the duration, timing, and intensity of outbreaks strongly and change the map of infectious diseases in the globe (45). Mosquito-transmitted parasitic or viral diseases are extremely climate-sensitive, as warming firstly shortens the pathogen incubation period and secondly shifts the geographic map of the vector. Similarly, water-warming following climate changes leads to a high incidence of waterborne infections. Recently, in Europe, eradicated diseases seem to be emerging due to the migration of population, for example, cholera, poliomyelitis, tick-borne encephalitis, and malaria (46).

The spread of epidemics is associated with natural climate disasters and storms, which seem to occur more frequently nowadays (47). Malnutrition and disequilibration of the immune system are also associated with the emerging infections affecting public health (48).

The Chikungunya virus “took the airplane” from the Indian Ocean to Europe, as outbreaks of the disease were registered in Italy (49) as well as autochthonous cases in France (50).

An increase in cryptosporidiosis in the United Kingdom and in the Czech Republic seems to have occurred following flooding (36, 51).

As stated previously, aerosols compounds are tiny in size and considerably affect the climate. They are able to dissipate sunlight (the albedo phenomenon) by dispersing a quarter of the sun's rays back to space and have cooled the global temperature over the last 30 years (52).

## Air Pollutants

The World Health Organization (WHO) reports on six major air pollutants, namely particle pollution, ground-level ozone, carbon monoxide, sulfur oxides, nitrogen oxides, and lead. Air pollution can have a disastrous effect on all components of the environment, including groundwater, soil, and air. Additionally, it poses a serious threat to living organisms. In this vein, our interest is mainly to focus on these pollutants, as they are related to more extensive and severe problems in human health and environmental impact. Acid rain, global warming, the greenhouse effect, and climate changes have an important ecological impact on air pollution (53).

### Particulate Matter (PM) and Health

Studies have shown a relationship between particulate matter (PM) and adverse health effects, focusing on either short-term (acute) or long-term (chronic) PM exposure.

Particulate matter (PM) is usually formed in the atmosphere as a result of chemical reactions between the different pollutants. The penetration of particles is closely dependent on their size (53). Particulate Matter (PM) was defined as a term for particles by the United States Environmental Protection Agency (54). Particulate matter (PM) pollution includes particles with diameters of 10 micrometers (μm) or smaller, called PM_10_, and extremely fine particles with diameters that are generally 2.5 micrometers (μm) and smaller.

Particulate matter contains tiny liquid or solid droplets that can be inhaled and cause serious health effects (55). Particles <10 μm in diameter (PM_10_) after inhalation can invade the lungs and even reach the bloodstream. Fine particles, PM_2.5_, pose a greater risk to health (6, 56) (Table 1).

**Table 1:** Penetrability according to particle size.

**Particle size**	**Penetration degree in human respiratory system**
>11 μm	Passage into nostrils and upper respiratory tract
7–11 μm	Passage into nasal cavity
4.7–7 μm	Passage into larynx
3.3–4.7 μm	Passage into trachea-bronchial area
2.1–3.3 μm	Secondary bronchial area passage
1.1–2.1 μm	Terminal bronchial area passage
0.65–1.1 μm	Bronchioles penetrability
0.43–0.65 μm	Alveolar penetrability

Multiple epidemiological studies have been performed on the health effects of PM. A positive relation was shown between both short-term and long-term exposures of PM_2.5_ and acute nasopharyngitis (56). In addition, long-term exposure to PM for years was found to be related to cardiovascular diseases and infant mortality.

Those studies depend on PM_2.5_ monitors and are restricted in terms of study area or city area due to a lack of spatially resolved daily PM_2.5_ concentration data and, in this way, are not representative of the entire population. Following a recent epidemiological study by the Department of Environmental Health at Harvard School of Public Health (Boston, MA) (57), it was reported that, as PM_2.5_ concentrations vary spatially, an exposure error (Berkson error) seems to be produced, and the relative magnitudes of the short- and long-term effects are not yet completely elucidated. The team developed a PM_2.5_ exposure model based on remote sensing data for assessing short- and long-term human exposures (57). This model permits spatial resolution in short-term effects plus the assessment of long-term effects in the whole population.

Moreover, respiratory diseases and affection of the immune system are registered as long-term chronic effects (58). It is worth noting that people with asthma, pneumonia, diabetes, and respiratory and cardiovascular diseases are especially susceptible and vulnerable to the effects of PM. PM_2.5_, followed by PM_10_, are strongly associated with diverse respiratory system diseases (59), as their size permits them to pierce interior spaces (60). The particles produce toxic effects according to their chemical and physical properties. The components of PM_10_ and PM_2.5_ can be organic (polycyclic aromatic hydrocarbons, dioxins, benzene, 1-3 butadiene) or inorganic (carbon, chlorides, nitrates, sulfates, metals) in nature (55).

Particulate Matter (PM) is divided into four main categories according to type and size (61) (Table 2).

**Table 2:** Types and sizes of particulate Matter (PM).

**Type**		**PM diameter [μm]**
Particulate contaminants	Smog	0.01–1
	Soot	0.01–0.8
	Tobacco smoke	0.01–1
	Fly ash	1–100
	Cement Dust	8–100
Biological Contaminants	Bacteria and bacterial spores	0.7–10
	Viruses	0.01–1
	Fungi and molds	2–12
	Allergens (dogs, cats, pollen, household dust)	0.1–100
Types of Dust	Atmospheric dust	0.01–1
	Heavy dust	100–1000
	Settling dust	1–100
Gases	Different gaseous contaminants	0.0001–0.01

*Gas contaminants* include PM in aerial masses.

*Particulate contaminants* include contaminants such as smog, soot, tobacco smoke, oil smoke, fly ash, and cement dust.

*Biological Contaminants* are microorganisms (bacteria, viruses, fungi, mold, and bacterial spores), cat allergens, house dust and allergens, and pollen.

*Types of Dust* include suspended atmospheric dust, settling dust, and heavy dust.

Finally, another fact is that the half-lives of PM_10_ and PM_2.5_ particles in the atmosphere is extended due to their tiny dimensions; this permits their long-lasting suspension in the atmosphere and even their transfer and spread to distant destinations where people and the environment may be exposed to the same magnitude of pollution (53). They are able to change the nutrient balance in watery ecosystems, damage forests and crops, and acidify water bodies.

As stated, PM_2.5_, due to their tiny size, are causing more serious health effects. These aforementioned fine particles are the main cause of the “haze” formation in different metropolitan areas (12, 13, 61).

### Ozone Impact in the Atmosphere

Ozone (O_3_) is a gas formed from oxygen under high voltage electric discharge (62). It is a strong oxidant, 52% stronger than chlorine. It arises in the stratosphere, but it could also arise following chain reactions of photochemical smog in the troposphere (63).

Ozone can travel to distant areas from its initial source, moving with air masses (64). It is surprising that ozone levels over cities are low in contrast to the increased amounts occuring in urban areas, which could become harmful for cultures, forests, and vegetation (65) as it is reducing carbon assimilation (66). Ozone reduces growth and yield (47, 48) and affects the plant microflora due to its antimicrobial capacity (67, 68). In this regard, ozone acts upon other natural ecosystems, with microflora (69, 70) and animal species changing their species composition (71). Ozone increases DNA damage in epidermal keratinocytes and leads to impaired cellular function (72).

Ground-level ozone (GLO) is generated through a chemical reaction between oxides of nitrogen and VOCs emitted from natural sources and/or following anthropogenic activities.

Ozone uptake usually occurs by inhalation. Ozone affects the upper layers of the skin and the tear ducts (73). A study of short-term exposure of mice to high levels of ozone showed malondialdehyde formation in the upper skin (epidermis) but also depletion in vitamins C and E. It is likely that ozone levels are not interfering with the skin barrier function and integrity to predispose to skin disease (74).

Due to the low water-solubility of ozone, inhaled ozone has the capacity to penetrate deeply into the lungs (75).

Toxic effects induced by ozone are registered in urban areas all over the world, causing biochemical, morphologic, functional, and immunological disorders (76).

The European project (APHEA2) focuses on the acute effects of ambient ozone concentrations on mortality (77). Daily ozone concentrations compared to the daily number of deaths were reported from different European cities for a 3-year period. During the warm period of the year, an observed increase in ozone concentration was associated with an increase in the daily number of deaths (0.33%), in the number of respiratory deaths (1.13%), and in the number of cardiovascular deaths (0.45%). No effect was observed during wintertime.

### Carbon Monoxide (CO)

Carbon monoxide is produced by fossil fuel when combustion is incomplete. The symptoms of poisoning due to inhaling carbon monoxide include headache, dizziness, weakness, nausea, vomiting, and, finally, loss of consciousness.

The affinity of carbon monoxide to hemoglobin is much greater than that of oxygen. In this vein, serious poisoning may occur in people exposed to high levels of carbon monoxide for a long period of time. Due to the loss of oxygen as a result of the competitive binding of carbon monoxide, hypoxia, ischemia, and cardiovascular disease are observed.

Carbon monoxide affects the greenhouses gases that are tightly connected to global warming and climate. This should lead to an increase in soil and water temperatures, and extreme weather conditions or storms may occur (68).

However, in laboratory and field experiments, it has been seen to produce increased plant growth (78).

### Nitrogen Oxide (NO_2_)

Nitrogen oxide is a traffic-related pollutant, as it is emitted from automobile motor engines (79, 80). It is an irritant of the respiratory system as it penetrates deep in the lung, inducing respiratory diseases, coughing, wheezing, dyspnea, bronchospasm, and even pulmonary edema when inhaled at high levels. It seems that concentrations over 0.2 ppm produce these adverse effects in humans, while concentrations higher than 2.0 ppm affect T-lymphocytes, particularly the CD8+ cells and NK cells that produce our immune response (81).It is reported that long-term exposure to high levels of nitrogen dioxide can be responsible for chronic lung disease. Long-term exposure to NO_2_ can impair the sense of smell (81).

However, systems other than respiratory ones can be involved, as symptoms such as eye, throat, and nose irritation have been registered (81).

High levels of nitrogen dioxide are deleterious to crops and vegetation, as they have been observed to reduce crop yield and plant growth efficiency. Moreover, NO_2_ can reduce visibility and discolor fabrics (81).

### Sulfur Dioxide (SO_2_)

Sulfur dioxide is a harmful gas that is emitted mainly from fossil fuel consumption or industrial activities. The annual standard for SO_2_ is 0.03 ppm (82). It affects human, animal, and plant life. Susceptible people as those with lung disease, old people, and children, who present a higher risk of damage. The major health problems associated with sulfur dioxide emissions in industrialized areas are respiratory irritation, bronchitis, mucus production, and bronchospasm, as it is a sensory irritant and penetrates deep into the lung converted into bisulfite and interacting with sensory receptors, causing bronchoconstriction. Moreover, skin redness, damage to the eyes (lacrimation and corneal opacity) and mucous membranes, and worsening of pre-existing cardiovascular disease have been observed (81).

Environmental adverse effects, such as acidification of soil and acid rain, seem to be associated with sulfur dioxide emissions (83).

### Lead

Lead is a heavy metal used in different industrial plants and emitted from some petrol motor engines, batteries, radiators, waste incinerators, and waste waters (84).

Moreover, major sources of lead pollution in the air are metals, ore, and piston-engine aircraft. Lead poisoning is a threat to public health due to its deleterious effects upon humans, animals, and the environment, especially in the developing countries.

Exposure to lead can occur through inhalation, ingestion, and dermal absorption. Trans- placental transport of lead was also reported, as lead passes through the placenta unencumbered (85). The younger the fetus is, the more harmful the toxic effects. Lead toxicity affects the fetal nervous system; edema or swelling of the brain is observed (86). Lead, when inhaled, accumulates in the blood, soft tissue, liver, lung, bones, and cardiovascular, nervous, and reproductive systems. Moreover, loss of concentration and memory, as well as muscle and joint pain, were observed in adults (85, 86).

Children and newborns (87) are extremely susceptible even to minimal doses of lead, as it is a neurotoxicant and causes learning disabilities, impairment of memory, hyperactivity, and even mental retardation.

Elevated amounts of lead in the environment are harmful to plants and crop growth. Neurological effects are observed in vertebrates and animals in association with high lead levels (88).

### Polycyclic Aromatic Hydrocarbons(PAHs)

The distribution of PAHs is ubiquitous in the environment, as the atmosphere is the most important means of their dispersal. They are found in coal and in tar sediments. Moreover, they are generated through incomplete combustion of organic matter as in the cases of forest fires, incineration, and engines (89). PAH compounds, such as benzopyrene, acenaphthylene, anthracene, and fluoranthene are recognized as toxic, mutagenic, and carcinogenic substances. They are an important risk factor for lung cancer (89).

### Volatile Organic Compounds(VOCs)

Volatile organic compounds (VOCs), such as toluene, benzene, ethylbenzene, and xylene (90), have been found to be associated with cancer in humans (91). The use of new products and materials has actually resulted in increased concentrations of VOCs. VOCs pollute indoor air (90) and may have adverse effects on human health (91). Short-term and long-term adverse effects on human health are observed. VOCs are responsible for indoor air smells. Short-term exposure is found to cause irritation of eyes, nose, throat, and mucosal membranes, while those of long duration exposure include toxic reactions (92). Predictable assessment of the toxic effects of complex VOC mixtures is difficult to estimate, as these pollutants can have synergic, antagonistic, or indifferent effects (91, 93).

### Dioxins

Dioxins originate from industrial processes but also come from natural processes, such as forest fires and volcanic eruptions. They accumulate in foods such as meat and dairy products, fish and shellfish, and especially in the fatty tissue of animals (94).

Short-period exhibition to high dioxin concentrations may result in dark spots and lesions on the skin (94). Long-term exposure to dioxins can cause developmental problems, impairment of the immune, endocrine and nervous systems, reproductive infertility, and cancer (94).

Without any doubt, fossil fuel consumption is responsible for a sizeable part of air contamination. This contamination may be anthropogenic, as in agricultural and industrial processes or transportation, while contamination from natural sources is also possible. Interestingly, it is of note that the air quality standards established through the European Air Quality Directive are somewhat looser than the WHO guidelines, which are stricter (95).

## Effect of Air Pollution on Health

The most common air pollutants are ground-level ozone and Particulates Matter (PM). Air pollution is distinguished into two main types:

*Outdoor pollution* is the ambient air pollution.

*Indoor pollution* is the pollution generated by household combustion of fuels.

People exposed to high concentrations of air pollutants experience disease symptoms and states of greater and lesser seriousness. These effects are grouped into short- and long-term effects affecting health.

Susceptible populations that need to be aware of health protection measures include old people, children, and people with diabetes and predisposing heart or lung disease, especially asthma.

As extensively stated previously, according to a recent epidemiological study from Harvard School of Public Health, the relative magnitudes of the short- and long-term effects have not been completely clarified (57) due to the different epidemiological methodologies and to the exposure errors. New models are proposed for assessing short- and long-term human exposure data more successfully (57). Thus, in the present section, we report the more common short- and long-term health effects but also general concerns for both types of effects, as these effects are often dependent on environmental conditions, dose, and individual susceptibility.

Short-term effects are temporary and range from simple discomfort, such as irritation of the eyes, nose, skin, throat, wheezing, coughing and chest tightness, and breathing difficulties, to more serious states, such as asthma, pneumonia, bronchitis, and lung and heart problems. Short-term exposure to air pollution can also cause headaches, nausea, and dizziness.

These problems can be aggravated by extended long-term exposure to the pollutants, which is harmful to the neurological, reproductive, and respiratory systems and causes cancer and even, rarely, deaths.

The long-term effects are chronic, lasting for years or the whole life and can even lead to death. Furthermore, the toxicity of several air pollutants may also induce a variety of cancers in the long term (96).

As stated already, respiratory disorders are closely associated with the inhalation of air pollutants. These pollutants will invade through the airways and will accumulate at the cells. Damage to target cells should be related to the pollutant component involved and its source and dose. Health effects are also closely dependent on country, area, season, and time. An extended exposure duration to the pollutant should incline to long-term health effects in relation also to the above factors.

Particulate Matter (PMs), dust, benzene, and O_3_ cause serious damage to the respiratory system (97). Moreover, there is a supplementary risk in case of existing respiratory disease such as asthma (98). Long-term effects are more frequent in people with a predisposing disease state. When the trachea is contaminated by pollutants, voice alterations may be remarked after acute exposure. Chronic obstructive pulmonary disease (COPD) may be induced following air pollution, increasing morbidity and mortality (99). Long-term effects from traffic, industrial air pollution, and combustion of fuels are the major factors for COPD risk (99).

Multiple cardiovascular effects have been observed after exposure to air pollutants (100). Changes occurred in blood cells after long-term exposure may affect cardiac functionality. Coronary arteriosclerosis was reported following long-term exposure to traffic emissions (101), while short-term exposure is related to hypertension, stroke, myocardial infracts, and heart insufficiency. Ventricle hypertrophy is reported to occur in humans after long-time exposure to nitrogen oxide (NO_2_) (102, 103).

Neurological effects have been observed in adults and children after extended-term exposure to air pollutants.

**Figure 1:**
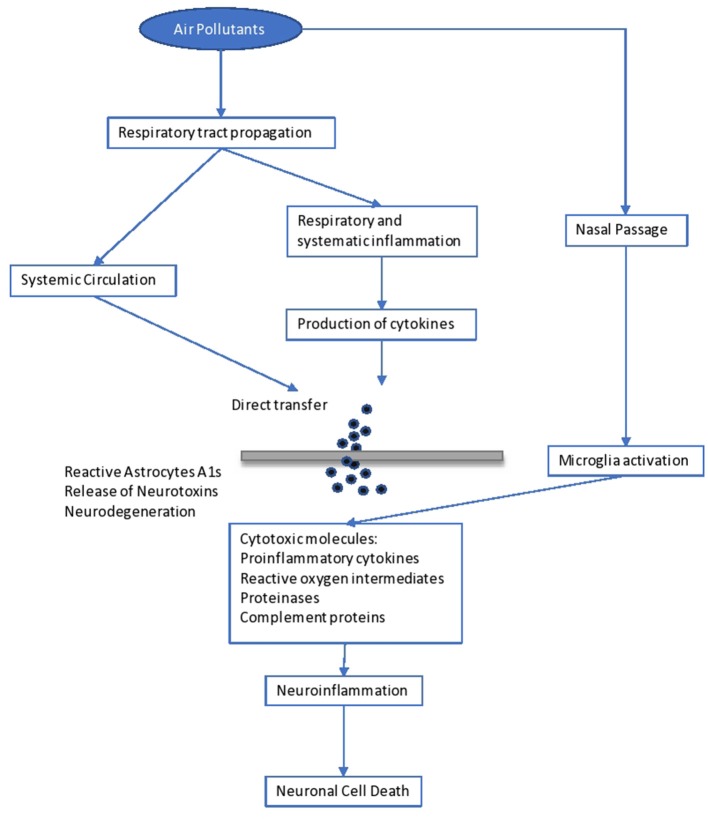
Impact of air pollutants on the brain.

Psychological complications, autism, retinopathy, fetal growth, and low birth weight seem to be related to long-term air pollution (83). The etiologic agent of the neurodegenerative diseases (Alzheimer's and Parkinson's) is not yet known, although it is believed that extended exposure to air pollution seems to be a factor. Specifically, pesticides and metals are cited as etiological factors, together with diet. The mechanisms in the development of neurodegenerative disease include oxidative stress, protein aggregation, inflammation, and mitochondrial impairment in neurons (104) (Figure 1).

Brain inflammation was observed in dogs living in a highly polluted area in Mexico for a long period (105). In human adults, markers of systemic inflammation (IL-6 and fibrinogen) were found to be increased as an immediate response to PNC on the IL-6 level, possibly leading to the production of acute-phase proteins (106). The progression of atherosclerosis and oxidative stress seem to be the mechanisms involved in the neurological disturbances caused by long-term air pollution. Inflammation comes secondary to the oxidative stress and seems to be involved in the impairment of developmental maturation, affecting multiple organs (105, 107). Similarly, other factors seem to be involved in the developmental maturation, which define the vulnerability to long-term air pollution. These include birthweight, maternal smoking, genetic background and socioeconomic environment, as well as education level.

However, diet, starting from breast-feeding, is another determinant factor. Diet is the main source of antioxidants, which play a key role in our protection against air pollutants (108). Antioxidants are free radical scavengers and limit the interaction of free radicals in the brain (108). Similarly, genetic background may result in a differential susceptibility toward the oxidative stress pathway (60). For example, antioxidant supplementation with vitamins C and E appears to modulate the effect of ozone in asthmatic children homozygous for the GSTM1 null allele (61). Inflammatory cytokines released in the periphery (e.g., respiratory epithelia) upregulate the innate immune Toll-like receptor 2. Such activation and the subsequent events leading to neurodegeneration have recently been observed in lung lavage in mice exposed to ambient Los Angeles (CA, USA) particulate matter (61). In children, neurodevelopmental morbidities were observed after lead exposure. These children developed aggressive and delinquent behavior, reduced intelligence, learning difficulties, and hyperactivity (109). No level of lead exposure seems to be “safe,” and the scientific community has asked the Centers for Disease Control and Prevention (CDC) to reduce the current screening guideline of 10 μg/dl (109).

It is important to state that impact on the immune system, causing dysfunction and neuroinflammation (104), is related to poor air quality. Yet, increases in serum levels of immunoglobulins (IgA, IgM) and the complement component C3 are observed (106). Another issue is that antigen presentation is affected by air pollutants, as there is an upregulation of costimulatory molecules such as CD80 and CD86 on macrophages (110).

As is known, skin is our shield against ultraviolet radiation (UVR) and other pollutants, as it is the most exterior layer of our body. Traffic-related pollutants, such as PAHs, VOCs, oxides, and PM, may cause pigmented spots on our skin (111). On the one hand, as already stated, when pollutants penetrate through the skin or are inhaled, damage to the organs is observed, as some of these pollutants are mutagenic and carcinogenic, and, specifically, they affect the liver and lung. On the other hand, air pollutants (and those in the troposphere) reduce the adverse effects of ultraviolet radiation UVR in polluted urban areas (111). Air pollutants absorbed by the human skin may contribute to skin aging, psoriasis, acne, urticaria, eczema, and atopic dermatitis (111), usually caused by exposure to oxides and photochemical smoke (111). Exposure to PM and cigarette smoking act as skin-aging agents, causing spots, dyschromia, and wrinkles. Lastly, pollutants have been associated with skin cancer (111).

Higher morbidity is reported to fetuses and children when exposed to the above dangers. Impairment in fetal growth, low birth weight, and autism have been reported (112).

Another exterior organ that may be affected is the eye. Contamination usually comes from suspended pollutants and may result in asymptomatic eye outcomes, irritation (112), retinopathy, or dry eye syndrome (113, 114).

## Environmental Impact of Air Pollution

Air pollution is harming not only human health but also the environment (115) in which we live. The most important environmental effects are as follows.

*Acid rain* is wet (rain, fog, snow) or dry (particulates and gas) precipitation containing toxic amounts of nitric and sulfuric acids. They are able to acidify the water and soil environments, damage trees and plantations, and even damage buildings and outdoor sculptures, constructions, and statues.

*Haze* is produced when fine particles are dispersed in the air and reduce the transparency of the atmosphere. It is caused by gas emissions in the air coming from industrial facilities, power plants, automobiles, and trucks.

*Ozone*, as discussed previously, occurs both at ground level and in the upper level (stratosphere) of the Earth's atmosphere. Stratospheric ozone is protecting us from the Sun's harmful ultraviolet (UV) rays. In contrast, ground-level ozone is harmful to human health and is a pollutant. Unfortunately, stratospheric ozone is gradually damaged by ozone-depleting substances (i.e., chemicals, pesticides, and aerosols). If this protecting stratospheric ozone layer is thinned, then UV radiation can reach our Earth, with harmful effects for human life (skin cancer) (116) and crops (117). In plants, ozone penetrates through the stomata, inducing them to close, which blocks CO_2_ transfer and induces a reduction in photosynthesis (118).

*Global climate change* is an important issue that concerns mankind. As is known, the “greenhouse effect” keeps the Earth's temperature stable. Unhappily, anthropogenic activities have destroyed this protecting temperature effect by producing large amounts of greenhouse gases, and global warming is mounting, with harmful effects on human health, animals, forests, wildlife, agriculture, and the water environment. A report states that global warming is adding to the health risks of poor people (119).

People living in poorly constructed buildings in warm-climate countries are at high risk for heat-related health problems as temperatures mount (119).

*Wildlife* is burdened by toxic pollutants coming from the air, soil, or the water ecosystem and, in this way, animals can develop health problems when exposed to high levels of pollutants. Reproductive failure and birth effects have been reported.

*Eutrophication* is occurring when elevated concentrations of nutrients (especially nitrogen) stimulate the blooming of aquatic algae, which can cause a disequilibration in the diversity of fish and their deaths.

Without a doubt, there is a critical concentration of pollution that an ecosystem can tolerate without being destroyed, which is associated with the ecosystem's capacity to neutralize acidity. The Canada Acid Rain Program established this load at 20 kg/ha/yr (120).

Hence, air pollution has deleterious effects on both soil and water (121). Concerning PM as an air pollutant, its impact on crop yield and food productivity has been reported. Its impact on watery bodies is associated with the survival of living organisms and fishes and their productivity potential (121).

An impairment in photosynthetic rhythm and metabolism is observed in plants exposed to the effects of ozone (121).

Sulfur and nitrogen oxides are involved in the formation of acid rain and are harmful to plants and marine organisms.

Last but not least, as mentioned above, the toxicity associated with lead and other metals is the main threat to our ecosystems (air, water, and soil) and living creatures (121).

## Discussion

In 2018, during the first WHO Global Conference on Air Pollution and Health, the WHO's General Director, Dr. Tedros Adhanom Ghebreyesus, called air pollution a “silent public health emergency” and “the new tobacco” (122).

Undoubtedly, children are particularly vulnerable to air pollution, especially during their development. Air pollution has adverse effects on our lives in many different respects.

Diseases associated with air pollution have not only an important economic impact but also a societal impact due to absences from productive work and school.

Despite the difficulty of eradicating the problem of anthropogenic environmental pollution, a successful solution could be envisaged as a tight collaboration of authorities, bodies, and doctors to regularize the situation. Governments should spread sufficient information and educate people and should involve professionals in these issues so as to control the emergence of the problem successfully.

Technologies to reduce air pollution at the source must be established and should be used in all industries and power plants. The Kyoto Protocol of 1997 set as a major target the reduction of GHG emissions to below 5% by 2012 (123). This was followed by the Copenhagen summit, 2009 (124), and then the Durban summit of 2011 (125), where it was decided to keep to the same line of action. The Kyoto protocol and the subsequent ones were ratified by many countries. Among the pioneers who adopted this important protocol for the world's environmental and climate “health” was China (3). As is known, China is a fast-developing economy and its GDP (Gross Domestic Product) is expected to be very high by 2050, which is defined as the year of dissolution of the protocol for the decrease in gas emissions.

A more recent international agreement of crucial importance for climate change is the Paris Agreement of 2015, issued by the UNFCCC (United Nations Climate Change Committee). This latest agreement was ratified by a plethora of UN (United Nations) countries as well as the countries of the European Union (126). In this vein, parties should promote actions and measures to enhance numerous aspects around the subject. Boosting education, training, public awareness, and public participation are some of the relevant actions for maximizing the opportunities to achieve the targets and goals on the crucial matter of climate change and environmental pollution (126). Without any doubt, technological improvements makes our world easier and it seems difficult to reduce the harmful impact caused by gas emissions, we could limit its use by seeking reliable approaches.

Synopsizing, a global prevention policy should be designed in order to combat anthropogenic air pollution as a complement to the correct handling of the adverse health effects associated with air pollution. Sustainable development practices should be applied, together with information coming from research in order to handle the problem effectively.

At this point, international cooperation in terms of research, development, administration policy, monitoring, and politics is vital for effective pollution control. Legislation concerning air pollution must be aligned and updated, and policy makers should propose the design of a powerful tool of environmental and health protection. As a result, the main proposal of this essay is that we should focus on fostering local structures to promote experience and practice and extrapolate these to the international level through developing effective policies for sustainable management of ecosystems.

## Author Contributions

All authors listed have made a substantial, direct and intellectual contribution to the work, and approved it for publication.

### Conflict of Interest

IM is employed by the company Delphis S.A. The remaining authors declare that the present review paper was conducted in the absence of any commercial or financial relationships that could be construed as a potential conflict of interest.

## References

[1] WHO Air Pollution. WHO. Available online at: http://www.who.int/airpollution/en/ (accessed October 5, 2019).

[2] Moores FC Climate change and air pollution: exploring the synergies and potential for mitigation in industrializing countries. Sustainability. (2009) 1:43–54. 10.3390/su1010043

[3] USGCRP (2009). Global Climate Change Impacts in the United States. In: Karl TR Melillo JM Peterson TC, editors. Climate Change Impacts by Sectors: Ecosystems. New York, NY: United States Global Change Research Program. Cambridge University Press.

[4] Marlon JR Bloodhart B Ballew MT Rolfe-Redding J Roser-Renouf C Leiserowitz A (2019). How hope and doubt affect climate change mobilization. Front. Commun. 4:20 10.3389/fcomm.2019.00020

[5] Eze IC Schaffner E Fischer E Schikowski T Adam M Imboden M. Long- term air pollution exposure and diabetes in a population-based Swiss cohort. Environ Int. (2014) 70:95–105. 10.1016/j.envint.2014.05.014 24912113

[6] Kelishadi R Poursafa P. Air pollution and non-respiratory health hazards for children. Arch Med Sci. (2010) 6:483–95. 10.5114/aoms.2010.14458 22371790 PMC3284061

[7] Manucci PM Franchini M Health effects of ambient air pollution in developing countries. Int J Environ Res Public Health. (2017) 14:1048 10.3390/ijerph14091048 PMC5615585 28895888

[8] Burden of Disease from Ambient and Household Air Pollution. Available online: http://who.int/phe/health_topics/outdoorair/databases/en/ (accessed August 15, 2017).

[9] Hashim D Boffetta P. Occupational and environmental exposures and cancers in developing countries. Ann Glob Health. (2014) 80:393–411. 10.1016/j.aogh.2014.10.002 25512155

[10] Guo Y Zeng H Zheng R Li S Pereira G Liu Q. The burden of lung cancer mortality attributable to fine particles in China. Total Environ Sci. (2017) 579:1460–6. 10.1016/j.scitotenv.2016.11.147 27913022

[11] Hou Q An XQ Wang Y Guo JP. An evaluation of resident exposure to respirable particulate matter and health economic loss in Beijing during Beijing 2008 Olympic Games. Sci Total Environ. (2010) 408:4026–32. 10.1016/j.scitotenv.2009.12.030 20542537

[12] Kan H Chen R Tong S. Ambient air pollution, climate change, and population health in China. Environ Int. (2012) 42:10–9. 10.1016/j.envint.2011.03.003 21440303

[13] Burroughs Peña MS Rollins A Environmental exposures and cardiovascular disease: a challenge for health and development in low- and middle-income countries. Cardiol Clin. (2017) 35:71–86. 10.1016/j.ccl.2016.09.001 27886791 PMC5129872

[14] Kankaria A Nongkynrih B Gupta S. Indoor air pollution in india: implications on health and its control. Indian J Comm Med. 39:203–7. 10.4103/0970-0218.143019 25364142 PMC4215499

[15] Parajuli I Lee H Shrestha KR Indoor air quality and ventilation assessment of rural mountainous households of Nepal. Int J Sust Built Env. (2016) 5:301–11. 10.1016/j.ijsbe.2016.08.003

[16] Saud T Gautam R Mandal TK Gadi R Singh DP Sharma SK Emission estimates of organic and elemental carbon from household biomass fuel used over the Indo-Gangetic Plain (IGP), India. Atmos Environ. (2012) 61:212–20. 10.1016/j.atmosenv.2012.07.030

[17] Singh DP Gadi R Mandal TK Saud T Saxena M Sharma SK Emissions estimates of PAH from biomass fuels used in rural sector of Indo-Gangetic Plains of India. Atmos Environ. (2013) 68:120–6. 10.1016/j.atmosenv.2012.11.042

[18] Dherani M Pope D Mascarenhas M Smith KR Weber M BN. Indoor air pollution from unprocessed solid fuel use and pneumonia risk in children aged under five years: a systematic review and meta-analysis. Bull World Health Organ. (2008) 86:390–4. 10.2471/BLT.07.044529 18545742 PMC2647443

[19] Kassomenos P Kelessis A Petrakakis M Zoumakis N Christides T Paschalidou AK Air Quality assessment in a heavily-polluted urban Mediterranean environment through Air Quality indices. Ecol Indic. (2012) 18:259–68. 10.1016/j.ecolind.2011.11.021

[20] Dockery DW Pope CA Xu X Spengler JD Ware JH Fay ME. An association between air pollution and mortality in six U.S. cities. N Engl J Med. (1993) 329:1753–9. 10.1056/NEJM199312093292401 8179653

[21] Schwela DH Köth-Jahr I Leitfaden für die Aufstellung von Luftreinhalteplänen [Guidelines for the Implementation of Clean Air Implementation Plans]. Landesumweltamt des Landes Nordrhein Westfalen. State Environmental Service of the State of North Rhine-Westphalia (1994).

[22] Newlands M Environmental Activism, Environmental Politics, and Representation: The Framing of the British Environmental Activist Movement. Ph.D. thesis. University of East London, United Kingdom (2015).

[23] NEPIS (National Service Center for Environmental Publications) US EPA (Environmental Protection Agency) (2017). Available online at: https://www.epa.gov/clean-air-act-overview/air-pollution-current-and-future-challenges (accessed August 15, 2017).

[24] NRC (National Research Council) Available online at: https://www.nap.edu/read/10728/chapter/1,2014 (accessed September 17, 2019).

[25] Bull A Traffic Congestion: The Problem and How to Deal With It. Santiago: Nationes Unidas, Cepal (2003).

[26] Spiegel J Maystre LY Environmental Pollution Control, Part VII - The Environment, Chapter 55, Encyclopedia of Occupational Health and Safety. Available online at: http://www.ilocis.org/documents/chpt55e.htm (accessed September 17, 2019).

[27] European Community Reports Assessment of the Effectiveness of European Air Quality Policies and Measures: Case Study 2; Comparison of the EU and US Air Quality Standards and Planning Requirements. (2004). Available online at: https://ec.europa.eu/environment/archives/cafe/activities/pdf/case_study2.pdf (accessed September 22, 2019).

[28] Gibson R Ward S Parties in the digital age; a review. J Represent Democracy. (2009) 45:87–100. 10.1080/00344890802710888

[29] Kaun A Uldam J Digital activism: after the hype. New Media Soc. (2017) 20:2099–106. 10.1177/14614448177319

[30] Sivitanides M Shah V The era of digital activism. In: 2011 Conference for Information Systems Applied Research(CONISAR) Proceedings Wilmington North Carolina, USA. Available online at: https://www.arifyildirim.com/ilt510/marcos.sivitanides.vivek.shah.pdf (accessed September 22, 2019).

[31] Möller L Schuetzle D Autrup H. Future research needs associated with the assessment of potential human health risks from exposure to toxic ambient air pollutants. Environ Health Perspect. (1994) 102(Suppl. 4):193–210. 10.1289/ehp.94102s4193 7529703 PMC1566924

[32] Jacobson MZ Jacobson PMZ Atmospheric Pollution: History, Science, and Regulation. Cambridge University Press (2002). p. 206 10.1256/wea.243.02

[33] Stover RH Flooding of soil for disease control. In: Mulder D, editor. Chapter 3. Developments in Agricultural and Managed Forest Ecology. Elsevier (1979). p. 19–28. Available online at: http://www.sciencedirect.com/science/article/pii/B9780444416926500094 10.1016/B978-0-444-41692-6.50009-4 (accessed July 1, 2019).

[34] Maipa V Alamanos Y Bezirtzoglou E Seasonal fluctuation of bacterial indicators in coastal waters. Microb Ecol Health Dis. (2001) 13:143–6. 10.1080/089106001750462687

[35] Bezirtzoglou E Dimitriou D Panagiou A Occurrence of *Clostridium perfringens* in river water by using a new procedure. Anaerobe. (1996) 2:169–73. 10.1006/anae.1996.0022

[36] Kjellstrom T Lodh M McMichael T Ranmuthugala G Shrestha R Kingsland S Air and Water Pollution: Burden and Strategies for Control. DCP, Chapter 43. 817–32 p. Available online at: https://www.dcp-3.org/sites/default/files/dcp2/DCP43.pdf (accessed September 17, 2017).

[37] Pathak RK Wang T Ho KF Lee SC Characteristics of summertime PM2.5 organic and elemental carbon in four major Chinese cities: implications of high acidity for water- soluble organic carbon (WSOC). Atmos Environ. (2011) 45:318–25. 10.1016/j.atmosenv.2010.10.021

[38] Bonavigo L Zucchetti M Mankolli H Water radioactive pollution and related environmental aspects. J Int Env Appl Sci. (2009) 4:357–63

[39] World Health Organization (WHO) Preventing Disease Through Healthy Environments: Towards an Estimate of the Environmental Burden of Disease. 1106 p. Available online at: https://www.who.int/quantifying_ehimpacts/publications/preventingdisease.pdf (accessed September 22, 2019).

[40] Stansfeld SA. Noise effects on health in the context of air pollution exposure. Int J Environ Res Public Health. (2015) 12:12735–60. 10.3390/ijerph121012735 26473905 PMC4626997

[41] Ethical Unicorn Everything You Need To Know About Aerosols & Air Pollution. (2019). Available online at: https://ethicalunicorn.com/2019/04/29/everything-you-need-to-know-about-aerosols-air-pollution/ (accessed October 4, 2019).

[42] Colbeck I Lazaridis M. Aerosols and environmental pollution. Sci Nat. (2009) 97:117–31. 10.1007/s00114-009-0594-x 19727639

[43] Incecik S Gertler A Kassomenos P Aerosols and air quality. Sci Total Env. (2014) 355, 488–9. 10.1016/j.scitotenv.2014.04.012 24834897

[44] D'Amato G Pawankar R Vitale C Maurizia L. Climate change and air pollution: effects on respiratory allergy. Allergy Asthma Immunol Res. (2016) 8:391–5. 10.4168/aair.2016.8.5.391 27334776 PMC4921692

[45] Bezirtzoglou C Dekas K Charvalos E. Climate changes, environment and infection: facts, scenarios and growing awareness from the public health community within Europe. Anaerobe. (2011) 17:337–40. 10.1016/j.anaerobe.2011.05.016 21664978

[46] Castelli F Sulis G. Migration and infectious diseases. Clin Microbiol Infect. (2017) 23:283–9. 10.1016/j.cmi.2017.03.012 28336382

[47] Watson JT Gayer M Connolly MA. Epidemics after natural disasters. Emerg Infect Dis. (2007) 13:1–5. 10.3201/eid1301.060779 17370508 PMC2725828

[48] Fenn B Malnutrition in Humanitarian Emergencies. Available online at: https://www.who.int/diseasecontrol_emergencies/publications/idhe_2009_london_malnutrition_fenn.pdf. (accessed August 15, 2017).

[49] Lindh E Argentini C Remoli ME Fortuna C Faggioni G Benedetti E. The Italian 2017 outbreak Chikungunya virus belongs to an emerging *Aedes albopictus*–adapted virus cluster introduced from the Indian subcontinent. Open Forum Infect Dis. (2019) 6:ofy321. 10.1093/ofid/ofy321 30697571 PMC6345083

[50] Calba C Guerbois-Galla M Franke F Jeannin C Auzet-Caillaud M Grard G Pigaglio L Decoppet A. Preliminary report of an autochthonous chikungunya outbreak in France, July to September 2017. Eur Surveill. (2017) 22:17-00647. 10.2807/1560-7917.ES.2017.22.39.17-00647 29019313 PMC5709952

[51] Menne B Murray V Floods in the WHO European Region: Health Effects and Their Prevention. Copenhagen: WHO; Weltgesundheits organisation, Regionalbüro für Europa (2013). Available online at: http://www.euro.who.int/data/assets/pdf_file/0020/189020/e96853.pdf (accessed 15 August 2017).

[52] Schneider SH. The greenhouse effect: science and policy. Science. (1989) 243:771–81. 10.1126/science.243.4892.771 17820424

[53] Wilson WE Suh HH. Fine particles and coarse particles: concentration relationships relevant to epidemiologic studies. J Air Waste Manag Assoc. (1997) 47:1238–49. 10.1080/10473289.1997.10464074 9448515

[54] US EPA (US Environmental Protection Agency) (2018). Available online at: https://www.epa.gov/pm-pollution/particulate-matter-pm-basics (accessed September 22, 2018).

[55] Cheung K Daher N Kam W Shafer MM Ning Z Schauer JJ Spatial and temporal variation of chemical composition and mass closure of ambient coarse particulate matter (PM10–2.5) in the Los Angeles area. Atmos Environ. (2011) 45:2651–62. 10.1016/j.atmosenv.2011.02.066

[56] Zhang L Yang Y Li Y Qian ZM Xiao W Wang X. Short-term and long-term effects of PM2.5 on acute nasopharyngitis in 10 communities of Guangdong, China. Sci Total Env. (2019) 688:136–42. 10.1016/j.scitotenv.2019.05.470. 31229811

[57] Kloog I Ridgway B Koutrakis P Coull BA Schwartz JD. Long- and short-term exposure to PM2.5 and mortality using novel exposure models, Epidemiology. (2013) 24:555–61. 10.1097/EDE.0b013e318294beaa 23676266 PMC4372644

[58] New Hampshire Department of Environmental Services Current and Forecasted Air Quality in New Hampshire. Environmental Fact Sheet (2019). Available online at: https://www.des.nh.gov/organization/commissioner/pip/factsheets/ard/documents/ard-16.pdf (accessed September 22, 2019).

[59] Kappos AD Bruckmann P Eikmann T Englert N Heinrich U Höppe P. Health effects of particles in ambient air. Int J Hyg Environ Health. (2004) 207:399–407. 10.1078/1438-4639-00306 15471105

[60] Boschi N (Ed.). Defining an educational framework for indoor air sciences education. In: Education and Training in Indoor Air Sciences. Luxembourg: Springer Science & Business Media (2012). 245 p.

[61] Heal MR Kumar P Harrison RM. Particles, air quality, policy and health. Chem Soc Rev. (2012) 41:6606–30. 10.1039/c2cs35076a 22660420

[62] Bezirtzoglou E Alexopoulos A Ozone history and ecosystems: a goliath from impacts to advance industrial benefits and interests, to environmental and therapeutical strategies. In: Ozone Depletion, Chemistry and Impacts. (2009). p. 135–45.

[63] Villányi V Turk B Franc B Csintalan Z Ozone Pollution and its Bioindication. In: Villányi V, editor. Air Pollution. London: Intech Open (2010). 10.5772/10047

[64] Massachusetts Department of Public Health Massachusetts State Health Assessment. Boston, MA (2017). Available online at: https://www.mass.gov/files/documents/2017/11/03/2017%20MA%20SHA%20final%20compressed.pdf (accessed October 30, 2017).

[65] Lorenzini G Saitanis C Ozone: A Novel Plant “Pathogen.” In: Sanitá di Toppi L Pawlik-Skowrońska B, editors. Abiotic Stresses in Plant Springer Link (2003). p. 205–29. 10.1007/978-94-017-0255-3_8

[66] Fares S Vargas R Detto M Goldstein AH Karlik J Paoletti E. Tropospheric ozone reduces carbon assimilation in trees: estimates from analysis of continuous flux measurements. Glob Change Biol. (2013) 19:2427–43. 10.1111/gcb.12222 23589473

[67] Harmens H Mills G Hayes F Jones L Norris D Fuhrer J Air Pollution and Vegetation. ICP Vegetation Annual Report 2006/2007. (2012)

[68] Emberson LD Pleijel H Ainsworth EA den Berg M Ren W Osborne S Ozone effects on crops and consideration in crop models. Eur J Agron. (2018) 100:19–34. 10.1016/j.eja.2018.06.002

[69] Alexopoulos A Plessas S Ceciu S Lazar V Mantzourani I Voidarou C Evaluation of ozone efficacy on the reduction of microbial population of fresh cut lettuce (*Lactuca sativa*) and green bell pepper (*Capsicum annuum*). Food Control. (2013) 30:491–6. 10.1016/j.foodcont.2012.09.018

[70] Alexopoulos A Plessas S Kourkoutas Y Stefanis C Vavias S Voidarou C. Experimental effect of ozone upon the microbial flora of commercially produced dairy fermented products. Int J Food Microbiol. (2017) 246:5–11. 10.1016/j.ijfoodmicro.2017.01.018 28187330

[71] Maggio A Fagnano M Ozone damages to mediterranean crops: physiological responses. Ital J Agron. (2008) 13–20. 10.4081/ija.2008.13

[72] McCarthy JT Pelle E Dong K Brahmbhatt K Yarosh D Pernodet N. Effects of ozone in normal human epidermal keratinocytes. Exp Dermatol. (2013) 22:360–1. 10.1111/exd.12125 23614745

[73] WHO Health Risks of Ozone From Long-Range Transboundary Air Pollution. Available online at: http://www.euro.who.int/data/assets/pdf_file/0005/78647/E91843.pdf (accessed August 15, 2019).

[74] Thiele JJ Traber MG Tsang K Cross CE Packer L. *In vivo* exposure to ozone depletes vitamins C and E and induces lipid peroxidation in epidermal layers of murine skin. Free Radic Biol Med. (1997) 23:365–91. 10.1016/S0891-5849(96)00617-X 9214574

[75] Hatch GE Slade R Harris LP McDonnell WF Devlin RB Koren HS. Ozone dose and effect in humans and rats. A comparison using oxygen- 18 labeling and bronchoalveolar lavage. Am J Respir Crit Care Med. (1994) 150:676–83. 10.1164/ajrccm.150.3.8087337 8087337

[76] Lippmann M. Health effects of ozone. A critical review. JAPCA. (1989) 39:672–95. 10.1080/08940630.1989.10466554 2659744

[77] Gryparis A Forsberg B Katsouyanni K Analitis A Touloumi G Schwartz J Acute effects of ozone on mortality from the “air pollution and health: a European approach” project. Am J Respir Crit Care Med. (2004) 170:1080–7. 10.1164/rccm.200403-333OC 15282198

[78] Soon W Baliunas SL Robinson AB Robinson ZW Environmental effects of increased atmospheric carbon dioxide. Climate Res. (1999) 13:149–64 10.1260/0958305991499694

[79] Richmont-Bryant J Owen RC Graham S Snyder M McDow S Oakes M Estimation of on-road NO2 concentrations, NO2/NOX ratios, and related roadway gradients from near-road monitoring data. Air Qual Atm Health. (2017) 10:611–25. 10.1007/s11869-016-0455-7 PMC6145484 30245748

[80] Hesterberg TW Bunn WB McClellan RO Hamade AK Long CM Valberg PA. Critical review of the human data on short-term nitrogen dioxide (NO_2_) exposures: evidence for NO2 no-effect levels. Crit Rev Toxicol. (2009) 39:743–81. 10.3109/10408440903294945 19852560

[81] Chen T-M Gokhale J Shofer S Kuschner WG. Outdoor air pollution: nitrogen dioxide, sulfur dioxide, and carbon monoxide health effects. Am J Med Sci. (2007) 333:249–56. 10.1097/MAJ.0b013e31803b900f 17435420

[82] US EPA Table of Historical SO_2_ NAAQS, Sulfur US EPA. Available online at: https://www3.epa.gov/ttn/naaqs/standards/so2/s_so2_history.html (accessed October 5, 2019).

[83] WHO Regional Office of Europe (2000). Available online at: https://euro.who.int/_data/assets/pdf_file/0020/123086/AQG2ndEd_7_4Sulfuroxide.pdf

[84] Pruss-Ustun A Fewrell L Landrigan PJ Ayuso-Mateos JL. Lead exposure. Comparative Quantification of Health Risks. World Health Organization. p. 1495–1542. Available online at: https://www.who.int/publications/cra/chapters/volume2/1495-1542.pdf?ua=1

[85] Goyer RA. Transplacental transport of lead. Environ Health Perspect. (1990) 89:101–5. 10.1289/ehp.9089101 2088735 PMC1567784

[86] National Institute of Environmental Health Sciences (NIH) Lead and Your Health. (2013). 1–4 p. Available online at: https://www.niehs.nih.gov/health/materials/lead_and_your_health_508.pdf (accessed September 17, 2019).

[87] Farhat A Mohammadzadeh A Balali-Mood M Aghajanpoor-Pasha M Ravanshad Y Correlation of blood lead level in mothers and exclusively breastfed infants: a study on infants aged less than six months. Asia Pac J Med Toxicol. (2013) 2:150–2.

[88] Assi MA Hezmee MNM Haron AW Sabri MYM Rajion MA. The detrimental effects of lead on human and animal health. Vet World. (2016) 9:660–71. 10.14202/vetworld.2016.660-671 27397992 PMC4937060

[89] Abdel-Shafy HI Mansour MSM A review on polycyclic aromatic hydrocarbons: source, environmental impact, effect on human health and remediation. Egypt J Pet. (2016) 25:107–23. 10.1016/j.ejpe.2015.03.011

[90] Kumar A Singh BP Punia M Singh D Kumar K Jain VK. Assessment of indoor air concentrations of VOCs and their associated health risks in the library of Jawaharlal Nehru University, New Delhi. Environ Sci Pollut Res Int. (2014) 21:2240–8. 10.1007/s11356-013-2150-7 24046229

[91] Molhave L Clausen G Berglund B Ceaurriz J Kettrup A Lindvall T Total Volatile Organic Compounds (TVOC) in Indoor Air Quality Investigations. Indoor Air. 7:225–240. 10.1111/j.1600-0668.1997.00002.x

[92] Gibb T Indoor Air Quality May be Hazardous to Your Health. MSU Extension. Available online at: https://www.canr.msu.edu/news/indoor_air_quality_may_be_hazardous_to_your_health (accessed October 5, 2019).

[93] Ebersviller S Lichtveld K Sexton KG Zavala J Lin Y-H Jaspers I. Gaseous VOCs rapidly modify particulate matter and its biological effects – Part 1: simple VOCs and model PM. Atmos Chem Phys Discuss. (2012) 12:5065–105. 10.5194/acpd-12-5065-2012 23457430 PMC3583354

[94] WHO (World Health Organization). Dioxins and Their Effects on Human Health. Available online at: https://www.who.int/news-room/fact-sheets/detail/dioxins-and-their-effects-on-human-health (accessed October 5, 2019).

[95] EEA (European Environmental Agency) Air Quality Standards to the European Union and WHO. Available online at: https://www.eea.europa.eu/themes/data-and-maps/figures/air-quality-standards-under-the

[96] Nakano T Otsuki T [Environmental air pollutants and the risk of cancer]. (Japanese). Gan To Kagaku Ryoho. (2013) 40:1441–5. 24231697

[97] Kurt OK Zhang J Pinkerton KE. Pulmonary health effects of air pollution. Curr Opin Pulm Med. (2016) 22:138–43. 10.1097/MCP.0000000000000248 26761628 PMC4776742

[98] Guarnieri M Balmes JR. Outdoor air pollution and asthma. Lancet. (2014) 383:1581–92. 10.1016/S0140-6736(14)60617-6 24792855 PMC4465283

[99] Jiang X-Q Mei X-D Feng D. Air pollution and chronic airway diseases: what should people know and do? J Thorac Dis. (2016) 8:E31–40. 26904251 10.3978/j.issn.2072-1439.2015.11.50 PMC4740163

[100] Bourdrel T Bind M-A Béjot Y Morel O Argacha J-F. Cardiovascular effects of air pollution. Arch Cardiovasc Dis. (2017) 110:634–42. 10.1016/j.acvd.2017.05.003 28735838 PMC5963518

[101] Hoffmann B Moebus S Möhlenkamp S Stang A Lehmann N Dragano N. Residential exposure to traffic is associated with coronary atherosclerosis. Circulation. (2007) 116:489–496. 10.1161/CIRCULATIONAHA.107.693622 17638927

[102] Katholi RE Couri DM. Left ventricular hypertrophy: major risk factor in patients with hypertension: update and practical clinical applications. Int J Hypertens. (2011) 2011:495349. 10.4061/2011/495349 21755036 PMC3132610

[103] Leary PJ Kaufman JD Barr RG Bluemke DA Curl CL Hough CL. Traffic- related air pollution and the right ventricle. the multi-ethnic study of atherosclerosis. Am J Respir Crit Care Med. (2014) 189:1093–100. 10.1164/rccm.201312-2298OC 24593877 PMC4098110

[104] Genc S Zadeoglulari Z Fuss SH Genc K. The adverse effects of air pollution on the nervous system. J Toxicol. (2012) 2012:782462. 10.1155/2012/782462 22523490 PMC3317189

[105] Calderon-Garciduenas L Azzarelli B Acuna H. Air pollution and brain damage. Toxicol Pathol. (2002) 30:373–89. 10.1080/01926230252929954 12051555

[106] Rückerl R Greven S Ljungman P Aalto P Antoniades C Bellander T. Air pollution and inflammation (interleukin-6, C-reactive protein, fibrinogen) in myocardial infarction survivors. Environ Health Perspect. (2007) 115:1072–80. 10.1289/ehp.10021 17637925 PMC1913563

[107] Peters A Veronesi B Calderón-Garcidueñas L Gehr P Chen LC Geiser M. Translocation and potential neurological effects of fine and ultrafine particles a critical update. Part Fibre Toxicol. (2006) 3:13–8. 10.1186/1743-8977-3-13 16961926 PMC1570474

[108] Kelly FJ. Dietary antioxidants and environmental stress. Proc Nutr Soc. (2004) 63:579–85. 10.1079/PNS2004388 15831130

[109] Bellinger DC. Very low lead exposures and children's neurodevelopment. Curr Opin Pediatr. (2008) 20:172–7. 10.1097/MOP.0b013e3282f4f97b 18332714

[110] Balbo P Silvestri M Rossi GA Crimi E Burastero SE. Differential role of CD80 and CD86 on alveolar macrophages in the presentation of allergen to T lymphocytes in asthma. Clin Exp Allergy J Br Soc Allergy Clin Immunol. (2001) 31:625–36. 10.1046/j.1365-2222.2001.01068.x 11359432

[111] Drakaki E Dessinioti C Antoniou C Air pollution and the skin. Front Environ Sci Eng China. (2014) 15:2–8. 10.3389/fenvs.2014.00011

[112] Weisskopf MG Kioumourtzoglou M-A Roberts AL Air pollution and autism spectrum disorders: causal or confounded? Curr Environ Health Rep. (2015) 2:430–9. 10.1007/s40572-015-0073-9 26399256 PMC4737505

[113] Mo Z Fu Q Lyu D Zhang L Qin Z Tang Q. Impacts of air pollution on dry eye disease among residents in Hangzhou, China: a case-crossover study. Environ Pollut. (2019) 246:183–9. 10.1016/j.envpol.2018.11.109 30543944

[114] Klopfer J. Effects of environmental air pollution on the eye. J Am Optom Assoc. (1989) 60:773–8. 2685084

[115] Ashfaq A Sharma P Environmental effects of air pollution and application of engineered methods to combat the problem. J Indust Pollut Control. (2012) 29.

[116] Madronich S de Gruijl F. Skin cancer and UV radiation. Nature. (1993) 366:23–9. 10.1038/366023a0 8232533

[117] Teramura A Effects of UV-B radiation on the growth and yield of crop plants. Physiol Plant. (2006) 58:415–27. 10.1111/j.1399-3054.1983.tb04203.x

[118] Singh E Tiwari S Agrawal M. Effects of elevated ozone on photosynthesis and stomatal conductance of two soybean varieties: a case study to assess impacts of one component of predicted global climate change. Plant Biol Stuttg Ger. (2009) 11(Suppl. 1):101–8. 10.1111/j.1438-8677.2009.00263.x 19778374

[119] Manderson L How global Warming is Adding to the Health Risks of Poor People. The Conversation. University of the Witwatersrand. Available online at: http://theconversation.com/how-global-warming-is-adding-to-the-health-risks-of-poor-people-109520 (accessed October 5, 2019).

[120] Ministers of Energy and Environment Federal/Provincial/Territorial Ministers of Energy and Environment (Canada), editor. The Canada-Wide Acid Rain Strategy for Post-2000. Halifax: The Ministers (1999). 11 p.

[121] Zuhara S Isaifan R The impact of criteria air pollutants on soil and water: a review. (2018) 278–84. 10.30799/jespr.133.18040205

[122] WHO. First WHO Global Conference on Air Pollution and Health. (2018). Available online at: https://www.who.int/airpollution/events/conference/en/ (accessed October 6, 2019).

[123] What is the Kyoto Protocol? UNFCCC. Available online at: https://unfccc.int/kyoto__protocol (accessed October 6, 2019).

[124] CopenhagenClimate Change Conference (UNFCCC). Available online at: https://unfccc.int/process-and-meetings/conferences/past-conferences/copenhagen-climate-change-conference-december-2009/copenhagen-climate-change-conference-december-2009 (accessed October 6, 2019).

[125] Durban Climate Change Conference,. UNFCCC (2011). Available online at: https://unfccc.int/process-and-meetings/conferences/past-conferences/copenhagen-climate-change-conference-december-2009/copenhagen-climate-change-conference-december-2009 (accessed October 6, 2019).

[126] Paris Climate Change Agreement,. (2016). Available online at: https://unfccc.int/process-and-meetings/the-paris-agreement/the-paris-agreement

